# Les pneumopathies nosocomiales en réanimation de CHU Hassan II de Fès

**DOI:** 10.11604/pamj.2015.22.285.7630

**Published:** 2015-11-23

**Authors:** Abdelkarim Shimi, Soumaya Touzani, Nabil Elbakouri, Brahim Bechri, Ali Derkaoui, Mohammed Khatouf

**Affiliations:** 1 Service de Réanimation Polyvalente A1, CHU Hassan II, Faculté de Médecine et de Pharmacie, Université Sidi Mohamed Ben Abdellah, Fez, Maroc

**Keywords:** Pneumopathies nosocomiale, réanimation, résistance bactérienne, nosocomial pneumonia, resuscitation, bacterial resistance

## Abstract

L'objectif principal de notre étude était d'identifier les bactéries incriminées dans la pneumopathie nosocomiale (PN) au service de réanimation A1 du CHU Hassan II de Fès, en vue d'en améliorer la prise en charge et de diminuer la morbi-mortalité associée. Il s'agit d'une étude rétrospective et descriptive, menée du 1er janvier 2012 au 31 décembre 2013. Seules les infections pulmonaires survenant au-delà de 48 heures de l'admission du patient au service de réanimation ont été incluses. L'incidence de la PN était de 11,2%. Les Bacilles à Gram négatif (BGN) étaient retrouvés dans 48,5% des cas, le *Staphylocoque Aureus* dans 21,21% des cas et le Klebsiella *Pneumoniae* était dans 10,7% des cas. Le taux de mortalité était de 48,33%. L’âge, la gravité de la pathologie sous jacente et le retard de l'instauration d'une antibiothérapie adaptée étaient considérées comme facteurs de mauvais pronostic. L’étude de la résistance aux antibiotiques, montre une multirésistance surtout pour les BGN, dont il faut tenir compte en mettant en place une stratégie de prévention active.

## Introduction

La pneumopathie nosocomiale (PN) est un problème de santé publique qui concerne tous les services hospitaliers et en particulier la réanimation. L'infection nosocomiale est préoccupante par sa fréquence, son incidence éventuelle sur le pronostic de l'affection initiale et par son coût. Les pneumopathies nosocomiales posent des problèmes spécifiques en milieu de réanimation et particulièrement lorsqu'elles se développent chez le malade ventilé. Elles sont alors de diagnostic difficile, les agents responsables sont souvent résistants et leur pronostic est grave [1]. Le but de ce travail est d'analyser le profil bactériologique des pneumopathies nosocomiales en précisant leur incidence, en identifiant les facteurs prédisposant et ceux qui influencent le pronostic.

## Méthodes

Ce travail rétrospectif a porté sur l'analyse des dossiers de malades hospitalisés dans le service de réanimation polyvalente A1 durant une année (du 1^er^ janvier 2012 au 31 décembre 2013). Nous n'avons pris en compte que les malades hospitalisés 48 heures ou plus. Seules les infections pulmonaires survenant plus de 48 heures après l'admission du patient au service sont prises en compte. Les critères diagnostiques des PN sont ceux du Centers for Disease Control (CDC) d'Atlanta [2]. La pneumopathie est considérée certaine devant: la présence d'une ou plusieurs opacités parenchymateuses radiologiques anormales, récentes et évolutives; l'identification d'un microorganisme isolé au prélèvement trachéal distal par cathétérisme protégé en l'absence d'antibiothérapie récente avec plus de 10^2^ unités dormants colonies/ml; au moins un des signes suivants: des secrétions trachéales purulentes d'apparition récente, une fièvre récente en l'absence d'autres causes ou une hémoculture positive en l'absence d'autres foyers.

La PN est considérée possible devant: des signes radiologiques d'une ou de plusieurs opacités parenchymateuses anormales, récentes et persistantes et au moins un des signes suivants: des expectorations ou des secrétions trachéales purulentes; une fièvre >38°C d'apparition récente ou une hypothermie; une hyperleucocytose >10 000 ou une leucopénie; une détérioration des échanges gazeux.

## Résultats

Durant la période d’étude, 535 malades ont été hospitalisés au service de réanimation A1 dont 70% étaient de sexe masculin (n = 375) avec un âge moyen de 40.33 ans avec des extrêmes allant de 10 à 86 ans.

Nous avons enregistré 60 pneumopathies nosocomiales, ce qui représente un taux de 11,2%. 26,67% des pneumopathies nosocomiales (n = 16) ont été diagnostiquées selon des critères cliniques, biologiques et radiologiques sans réalisation de PDP en raison d'une urgence à instaurer un traitement antibiotique ou d'une plage horaire inadaptée (week-end). Sur les 52 PDP réalisés, 44 sont revenus positifs (84,62%). Le délai d'apparition de la PN est situé entre 03 jours et 26 jours d'hospitalisation avec une moyenne de 6,98 jours. La PN était précoce dans 45% des cas (27 cas) et tardive dans 55% des cas (33 cas).

Le motif d'admission était réparti comme suit: 30% des patients avaient un traumatisme crânien grave, 15% un accident vasculaire cérébral hémorragique, 13,33% un polytraumatisme grave et 10% un état de mal épileptique. La durée moyenne d'hospitalisation chez les malades infectés était de 18,5 jours contre 11 jours chez les malades non infectés. La durée moyenne de ventilation mécanique chez les malades infectés était de 10, 46 jours alors qu'elle était de 6,6 jours chez les malades non infectés. Les patients ayant une comorbidité représentaient 43,33% des malades atteints de PN alors qu'ils représentaient 32% des malades non infectés. La durée moyenne de la ventilation des patients atteints de PN était de 10,46 j alors que celle des patients non infectés était de 6,60 j.

L'analyse des facteurs pouvant influencer la survenue de la pneumopathie chez nos patients a permis d'identifier comme facteurs de risque: l’âge, la durée de la ventilation mécanique, la durée de séjour en réanimation, l'existence d'une comorbidité et la réintubation qui multiple par 2 le risque de la survenue d'une PN. La nature de la pathologie initiale s'est avérée un facteur qui peut prédisposer à la PN puisque les patients hospitalisés pour traumatisme crânien ou AVC hémorragique ont développé plus de PN (30% et 15%).

Les germes responsables des PN sont représentés dans le Tableau 1 il s'agit de bacilles à Gram négatif (BGN) dans 48,5% des cas et de staphylocoque aureus dans 21,21% des cas. Les PN étaient secondaires à un seul germe dans 55% des cas, d'une infection à deux germes dans 36% des cas et à 3 germes dans 9% des cas. La résistance des principaux germes aux antibiotiques au cours des PN est représentée dans le Tableau 2. Le taux de mortalité globale était de 24% alors que celui des malades atteints de PN était de 48,33% (29 cas). L'analyse des facteurs qui peuvent influencer la mortalité a permis d'identifier comme facteurs pronostiques: l’âge, la gravité de la pathologie sous jacente et le retard de l'instauration d'une antibiothérapie.

**Tableau 1:** Principaux germes responsable des pneumopathies nosocomiales

Germes	nombres	%
Pseudomonas aeroginosa	17	25,77
Staphylococcus aureus	14	21,21
Acinetobacter baumanii	10	15,16
Klebsiella pneumoniae	07	10,7
Haemophilus influenzae	05	07,57
Streptococcus pneumoniae	04	06,06
Serratia marcescens	04	06.06
Proteus mirabilis	02	03,03
E. coli	01	01,51
Morganella morgani	01	01,51
Klebsiella oxytoca	01	01,51
Total	66+	100

+ le total des germes recensés est supérieur à celui des infections car on peut isoler plusieurs germes chez le même malade

**Tableau 2:** Pourcentages de résistances des principaux germes aux antibiotiques

Germes					
	P. Aeruginosa	A. baumannii	K. pneumonia	S. aureus	H. influenzae
Nombre	17	10	07	14	05
Amoxicilline	--	--	100	85,51	30
Am + Ac.clav	--	--	71,43	21,43	00
Ticarcilline	52,94	80	57,14	---	---
Ceftazidime	17,62	90	28,57	28,57	10
Imipénem	17,62	30	14,28	---	--
Gentamycine	23,53	--	---	7,14	80
Amikacine	5,88	10	00	---	00
Ciprofloxacine	5,88	80	14,28	21,43	00
Triméthoprime sulfamétoxazol	82,35	--	14,28	71,43	30
Aztreonam	58,82	90	- --	---	--
Colistine	5,88	00	- --	--	--
Fosfomycine	23,53	----	----	-----	---

## Discussion

L'incidence des PN est très variable d'une étude à l'autre [3]. Cette variabilité est la conséquence d'une hétérogénéité des séries qui différent aussi bien dans la définition des critères diagnostiques que dans la provenance des patients. En réanimation, les PN occupent le premier rang avec un taux qui varie de 9% à 30% [4] voire 50% dans une l’étude de Diaza et al [5]. Dans notre série, l'incidence de PN était de 11,2%. Cette différence est sans doute liée en partie à la variation des procédés invasifs et des moyens diagnostiques d'un service à l'autre [6]; aux types de malades (terrain et pathologies nécessitant l'hospitalisation) et à la nature des études (calcul d'incidence ou de prévalence).

De nombreux facteurs, augmentant le risque de développer une PN, ont été identifiés mais le facteur essentiel semble être la ventilation mécanique [7]. Cette dernière augmente le risque par 4 et même par 21 [8]. Dans notre série, 16,75% des patients ventilés ont développé une PN. Dans la littérature, le taux de PN chez les patients ventilés est de 21.6% pour Chevret [9] et 27,7% pour Kollef [10].

La durée de la ventilation mécanique est un autre facteur de risque déterminant [8]. Le risque augmente de 1 à 3% pour chaque jour de ventilation [11]. Il est de 6,5% à 10 jours, de 19% à 20 jours jusqu’à 69% à 30 jours. Dans notre série, la durée moyenne de ventilation avant l'acquisition de la pneumopathie nosocomiale était de 6,60 jours avec des extrêmes allant de 3 jours à 26 jours. Les autres facteurs de risque soulevés dans la littérature sont l’âge > 70 ans [3], le score de gravité à l'admission [10], l'existence d'une bronchopneumopathie chronique, l’état de santé antérieur, une inhalation évidente, un trouble de la vigilance [10], une chirurgie thoracique ou sus méso colique urgente, une extubation intempestive ou une ré intubation [3], l'utilisation des médicaments inhibant l'acidité gastrique [8], la position déclive [10], un état de choc, les explorations endoscopiques bronchiques, un traumatisme thoracique et l'antibiothérapie préalable [10].

Plusieurs études ont permis d'identifier les germes responsables. Quels que soient les prélèvements utilisés, on retrouve en tête le *staphylocoque doré* et les germes à gram négatif. Les bacilles à Gram négatif sont responsable de plus de 60% des PN [12]. Dans les études les plus récentes, l’*Acinetobacter baumanii* est le germe le plus fréquemment isolé [13–16]. Dans notre étude, les BGN sont incriminés dans 48,5% des cas et ils sont dominés par le *Pseudomonas aeruginosa*. Les principaux germes retrouvés dans la littérature sont représentés dans le Tableau 3.

**Tableau 3:** Principaux germes responsables des PN dans la littérature

	Haond	Kollef	Chevret	Chastre	Medell	Notre serie
Staphylococcus aureus	30,7	27,9	24	08	20 ,8	21,21
P.aeruginosa	30,70	26,2	22,3	24,4	44,2	25,70
Acinetobacter baumannii	06,50		05,3	7,9	68,8	15,16
H.influenzae	12,8	6,5	07,4	20,4	--	07,57
K.pneumoniae	2.5	8,2	1	14,1	15,6	10,70

Plusieurs facteurs peuvent influencer l’étiologie bactérienne et la nature du germe. Le facteur essentiel est la durée de séjour à l'hôpital avant l'acquisition de la PN. Ce facteur va différencier ces dernières en 2 catégories [17]: les PN précoces qui surviennent avant le 5ème jour d'hospitalisation. Les germes retrouvés dans ce contexte sont ceux du patient (*Streptococcus pneumonae, Hemophilus influenzae, Staphylococcus aureus méthi-S, Escherchia Coli*) avec comme facteur de risque l'existence de trouble de la vigilance avec altération du réflexe de déglutition et de la toux. Dans notre série, on a noté 27 cas de pneumopathies précoces (45%) avec comme germes isolés: *Staphylococcus aureus dans 14 cas, Haemophilus infleunzae* dans 5 cas et le*streptocoque pneumonae* dans 4 cas; les PN tardives qui surviennent après le 5^ème^ jour d'hospitalisation. Elles sont liées à des germes hospitaliers multirésistants (*Pseudomonas aeruginosa, Acinetobacter baumani, Enterobacter*). Dans notre étude, la PN était tardive dans 55% des cas. Les germes isolés étaient le*Pseudomonas aeruginosa* dans 17 cas, l’*Acinetobacter baumani* dans 10 cas et le *Klebsiella Pneumoniae* chez 7 patients.

Les pneumopathies polymicrobiennes ne sont pas exceptionnelles. Une étude conduite par Combes et al sur 124 patients présentant une PN retrouve 48% d'infections poly microbiennes, sans différence d’épidémiologie ni d’évolution par rapport aux patients présentant une pneumopathie monomicrobienne [18]. Dans notre étude, on a isolé 2 à 3 germes dans 45% des PN. Le *Pseudomonas aeruginosa* (25,77% des cas), le *Staphylococcus Aureus* (21,21% des cas) et l’*Acinetobacter baumani* (15,16% des cas) sont les germes les plus fréquemment retrouvés, suivis des entérobactéries (*Klebsiella pneumoniae* et *E.coli*) contrairement aux données habituelles de la littérature qui rapportent où le *S.aureus* vient en premier suivi du *Pseudomonas aeruginosa* et des entérobactéries [19].

Le problème de résistance majeur se pose avec le *Pseudomonas aeruginosa* qui est naturellement résistant à de nombreux antibiotiques et qui acquière chaque jour de nouvelles résistances. Pour les entérobactéries, la résistance est due à la sécrétion d'une β-lactamase à spectre élargi. Le *Staphylococcus Aureus* a été isolé dans 21,21% des cas et il était résistant à la méthicilline chez un seul patient (7,14%).

La mortalité observée dans les séries de PN chez les malades ventilés est élevée variant de 20% dans l’étude de Craven [20] à 71% dans la série de Fagon [21]. Dans notre série, la mortalité chez les patients infectés était de 48,33%. Cette grande variation de la mortalité selon les séries est sans doute en rapport avec la présence ou non de facteurs qui influencent la mortalité surtout un état antérieur altéré ou une pathologie sous jacente grave [20, 21]. Chez nos patients, on a constaté que le mauvais pronostic était en rapport avec la gravité de la pathologie initiale et l'existence de co-morbidités.

Dans les services de réanimation, les pneumopathies nosocomiales sont responsables d'une augmentation de la charge de travail. Chez nos patients, la PN est responsable d'une prolongation de la durée de ventilation de 5 jours en moyenne et de la durée d'hospitalisation de 7 jours; ce qui a pour conséquence une aggravation du pronostic. La ventilation mécanique constitue le facteur favorisant majeur.

## Conclusion

L'instauration de la ventilation mécanique chez tout patient de réanimation doit s'accompagner d'une prudence majeure avec comme règles nécessaires: une asepsie rigoureuse lors des manipulations, des soins nasopharyngés fréquents en évitant toute situation pourvoyeuse de risque de contamination. L'amélioration du pronostic de ces patients passe obligatoirement par l'instauration d'une antibiothérapie adaptée sans retard et de façon à éviter la sélection des germes multi résistants.
